# Distinguishing laboratory characteristics in giant cell arteritis: a real-world retrospective cohort study

**DOI:** 10.1007/s10792-023-02829-5

**Published:** 2023-08-29

**Authors:** Raghav Goel, Eiman Usmani, Stephen Bacchi, Sumu Simon, Weng Onn Chan

**Affiliations:** 1 Royal Adelaide Hospital, Adelaide, SA 5000 Australia; 2 Department of Ophthalmology and Visual Sciences, University of Adelaide, Adelaide, SA Australia

**Keywords:** GCA, Retrospective study, GCA suspect, ESR, CRP

## Abstract

**Background:**

Untreated Giant Cell Arteritis (GCA) has the potential to cause serious complications such as vision loss. Appropriate initial assessment by General Practitioners, early treatment and specialist referral are therefore essential in reducing morbidity. However, lack of awareness around the range of presentations can lead to a delay in diagnosis.

**Objective:**

We aim to evaluate the discriminative diagnostic performance of laboratory characteristics associated with GCA in our population over a period of 18 months.

**Discussion:**

This is a real-world retrospective review of patients referred to ophthalmology services with concern for GCA. The pre-test probability of a patient referred with suspected GCA was 13.9% to have GCA, highlighting the need for specialist referrals to continue. White Cell Count (*p* = 0.01), Platelet Count (*p* = 0.02), Erythrocyte sedimentation rate (*p* = 0.004) and C-reactive protein (*p* = 0.002) were significantly different between GCA and non-GCA cases. Moreover, this study demonstrates that absolute neutrophil count (*p* = 0.02) can be a useful parameter in initial investigations for GCA.

## Introduction

General practitioners (GPs) are usually the first point of contact for patients with suspected giant cell arteritis (GCA). However, the diagnosis of GCA remains a challenge within the primary care setting due to the non-specific nature of the disease [1, 2]. Diagnostic delay is reported as 8 weeks or longer on average [1]. If left untreated, GCA can cause blindness, stroke, or other complications such as aortic aneurysm, dissection, and rupture. Additionally, the incidence of thromboembolic events in GCA is the highest within the first year of diagnosis [3]. Therefore, to facilitate timely treatment and reduce complications, prompt recognition and diagnosis of GCA are required.

The laboratory investigations can provide a vital clue to clinch the diagnosis. Typically, the erythrocyte sedimentation rate (ESR), C-reactive protein (CRP) and platelet counts are elevated at diagnosis. Previous studies have shown moderate sensitivities of ESR, CRP and platelets (65.5%, 66.9% and 71.2%, respectively) as stand-alone tests in the diagnosis of GCA [4, 5]. However, a combination of these inflammatory markers may provide improved diagnostic utility.

Recent data have highlighted the role of leukocytes such as neutrophils and monocytes in the pathogenesis of GCA [6, 7]. Persistently elevated counts of neutrophils throughout the course of the disease have rarely been documented [6]. Furthermore, there is no consensus in the literature on the optimal length of a temporal artery biopsy (TAB) specimen and traditionally a longer segment (> 15 mm) has been suggested to increase the diagnostic yield [8].

Thus, in the current study, we utilized full blood count to evaluate the discriminative diagnostic performance of laboratory characteristics of GCA in our population, so that we may better aid diagnosis within the primary care setting. We also explored the effect of TAB length on the outcomes of the biopsy results.

## Methods

This is a real-world retrospective cohort study. Demographic and laboratory results of patients referred to the Royal Adelaide and Queen Elizabeth Hospital ophthalmology outpatient clinic by General Practitioners, with concerns for GCA, were reviewed for the years 2020–2021. Subsequent clinic notes were followed up for a period of at least 6 months. All cases having overlap disease with polymyalgia rheumatica (PMR) and other vasculitides were excluded from the current study (*n* = 2). The study received Central Adelaide Local Health Network Human Research Ethics Committee (CALHN HREC) approval [Reference number: 14372] and adhered to the tenets of the Declaration of Helsinki.

### Diagnostic criteria used for GCA

A diagnosis of GCA was supported in clinic using the American College of Rheumatology (ACR) classification criteria (1990) [9]:

- Age at onset ≥ 50 years
- New headache
- Temporal artery abnormality such as tenderness to palpation or decreased pulsation
- Erythrocyte sedimentation rate ≥ 50 mm/h
- Abnormal artery biopsy showing vasculitis with mononuclear cell or granulomatous inflammation, usually with giant cell infiltrate

However, the diagnosis was made pragmatically by the decision of the treating specialist ophthalmologist and rheumatologist. This decision was reviewed and confirmed at 6 months. Tests such as raised CRP and platelets, non-compressible halo sign on Ultrasound (US) and Positron Emission Tomography (PET) activity increased probability of diagnosis as per the British Society of Rheumatology (BSR) guidelines [10]. Additional symptoms that raised high suspicion of GCA included sudden or transient vision loss, morning stiffness in shoulder or neck and jaw claudication. Giant cell arteritis probability score (GCAPS) was used to stratify risk [11].

Both cranial and extracranial cases were involved in the current study. The steroids used for GCA positive cases were Intravenous Methylprednisolone for 3 days followed by an oral taper. The duration varied for each patient given their constituent symptoms and reactions.

### Recognition of GCA in primary care

Referrals made by GPs were based on clinical features of the disease. Symptoms that raised suspicion of GCA included blurry vision, diplopia, sudden or transient vision loss, morning stiffness in shoulder or neck, jaw claudication, temporal headache or scalp tenderness. Eighty per cent of the referrals made to our clinic had visual symptoms of the disease.

### Statistical analysis

To examine for differences between GCA and non-GCA cases, Wilcoxon rank-sum tests were used to analyse initial laboratory results. The laboratory analysis was performed prior to the initiation of steroids and results sent to us along with the referral where applicable. When analysing each laboratory characteristic, if a case did not have that test result or if steroids were commenced prior to referral (without taking bloods; *n* = 2), then the case was excluded from the analysis of that outcome. Alpha was set at 0.05.

## Results

The results of the data collection method yielded 101 cases in which GCA was suspected. Of these, there were 14 cases of GCA (13.9%; 8 males, 6 females) and 87 that were non-GCA (86.1%). When comparing the laboratory tests between these groups, there were several results that had statistically significant differences (Table 1). Namely age (*p* = 0.046), white cell count (WCC) (*p* = 0.01), absolute neutrophil count (*p* = 0.02), platelet count (*p* = 0.02), ESR (*p* = 0.004) and CRP (*p* = 0.002) were different between GCA and non-GCA cases, respectively. Haemoglobin, mean cell volume and absolute lymphocyte count were not statistically different between the two groups. These results were age-adjusted prior to being reported.

**Table 1:** Characteristics of the GCA-confirmed and non-GCA cases in query GCA cohort

	GCA-confirmed cases	Non-GCA cases	Significance level
Number	14	87	
Gender (male/female)	8/6	30/57	*P* = 0.10
Mean age (years)	76.5 ± 9.59	70.3 ± 12.08	*P* = 0.046
Neutrophil count (× 10^9^/L)	7.4 ± 2.56	5.8 ± 2.19	*P* = 0.02
Mean WCC (× 10^9^/L)	10.2 ± 2.47	8.4 ± 2.22	*P* = 0.01
Platelet count (× 10^9^/L)	351.1 ± 117.57	275.9 ± 75.14	*P* = 0.02
Mean ESR (mm/hr)	70.9 ± 41.05	38.0 ± 33.53	*P* = 0.004
Mean CRP (mg/L)	54.3 ± 65.59	9.9 ± 16.17	*P* = 0.002

In the subgroup analyses of cases that were diagnosed as GCA (*n* = 14), there was no statistically significant difference in the laboratory parameters between the temporal artery biopsy (TAB) positive (*n* = 6) and negative (*n* = 6) cases. Two people had bilateral TABs in this subset. The two cases that did not have a TAB were excluded from this subgroup analysis.

When GCA cases that were temporal artery biopsy positive were compared to suspected GCA cases, laboratory results that were statistically significantly different were neutrophils (*p* = 0.033), platelets (*p* = 0.017), ESR (*p* = 0.031) and CRP (*p* = 0.00073). When GCA cases that were temporal artery biopsy negative were compared to suspected GCA cases, laboratory results that were statistically significantly different were ESR (*p* = 0.048). The specificity and sensitivity of absolute neutrophil count with regards to the TAB were 83% and 50%, respectively.

The mean length of the TAB-positive samples was 20.17 ± 10.19 mm as compared to 16.00 ± 7.80 mm for the TAB-negative samples (*p* = 0.376). At a biopsy length of < 15 mm, 33.3% (2/6) of the cases were TAB-positive. The number of TAB-positive cases increased to 66.7% (4/6) at a biopsy length of > 15 mm. The percentage of TAB-negative cases was 66.7% (4/6) at a biopsy length of < 15 mm but decreased to 33.3% (2/6) at a biopsy length of > 15 mm (p = 0.567). However, these did not reach statistical significance.

## Discussion

In this real-world retrospective cohort study, we found that the pre-test probability of a patient referred with suspected GCA (although not necessarily requiring a TAB) being found to have GCA is 13.9%. This highlights the need for ongoing specialist referrals with suspected GCA cases from general practitioners. Moreover, we found a significant difference in the absolute neutrophil count, in addition to WCC, platelet count, ESR and CRP, between GCA and suspected GCA cases.

Traditional serum inflammatory biomarkers such as ESR, CRP and platelets have established roles in the assessment of patients with suspected GCA [4, 5]. However, absolute neutrophil count has rarely been reported as a diagnostic marker [6, 12]. A recent study profiling peripheral blood leukocytes in biopsy-positive GCA patients demonstrated an elevated neutrophil presence throughout the course of the disease [6]. Other studies have suggested neutrophil-to-lymphocyte ratio (NLR) as a significant predictor of a positive biopsy in patients with suspected GCA [13]. Our study further highlights a statistically significant increase in the absolute neutrophil count within the GCA-confirmed cases (*p* = 0.02), which reinforces the involvement of neutrophils in the pathogenesis of GCA. However, the lack of difference in haemoglobin in this study is in contrast to other studies that have proposed that 20 to 50% of patients with GCA have normochromic normocytic anaemia [14]. Therefore, composite scores involving multiple laboratory parameters may improve the performance of such individual tests, although further research with large cohorts is required to have clinical validity.

While there is no consensus on the optimal length of a temporal artery biopsy specimen, a longer segment has previously been suggested to avoid a false negative GCA diagnosis due to the segmental nature of the disease. Specifically, some studies have proposed a 15 mm cut-off point [8]. “While the number of TAB-positive cases increased to 66.7% (from 33.3%) with >15 mm length segment, it was found to be not a statistically siginificant different in the mean length between TAB-positive and TAB-negative samples”. Nonetheless, these results are in keeping with recent data which highlight that a longer specimen length is not associated with a greater diagnostic yield [15]. The results depend more on adequate temporal artery histopathological sections and technical processing in the laboratory [16].

The current guidelines suggest prompt initiation of high-dose glucocorticoids when GCA is suspected combined with an urgent specialist referral for diagnostic confirmation [10]. Previous surveys have shown that up to one-third of primary care providers do not routinely initiate treatment routinely in suspected GCA cases [2]. Additionally, barriers to timely referral include variations in specialist referral pathways along with delays in accessing specialist care [2]. In the current study, two patients were on more than a month of steroid therapy prior to referral to our clinic. This may decrease the diagnostic yield of TAB as it is valuable within 4 weeks of starting high-dose corticosteroids and highest within the first 2 weeks [17].

We encourage the primary care providers to commence corticosteroids if tertiary care is not accessible immediately to prevent threatening complications of GCA. If accessible, they should be immediately referred to the nearest tertiary centre with specialist services to treat and investigate GCA. Symptoms that should raise high suspicion of GCA include sudden vision loss, morning stiffness in shoulder or neck, jaw claudication, new temporal headache, or scalp tenderness. Additionally, a rise in the inflammatory markers as described in the current study should also raise suspicion of the diagnosis.

There are limitations of the current study due to the retrospective nature of the design. For instance, some results did not reach significance due to our small sample size. Additionally, unequal sample sizes between the GCA-confirmed and non-GCA cases may have led to a decrease in statistical power of the results.

## Conclusion

In conclusion, this real-world retrospective study in our population has shown that 1 in 9 referrals of suspected GCA from primary care providers were found to be true. This potentiates the need for referrals to continue by primary care providers given the severe complications of GCA. Moreover, this study demonstrates that absolute neutrophil count can be useful in initial investigations for GCA in addition to WCC, ESR, CRP and platelet levels. These laboratory tests should be performed at baseline with an immediate specialist referral.

## References

[1] Prior JA, Ranjbar H, Belcher J (2017). Diagnostic delay for giant cell arteritis: a systematic review and meta-analysis. BMC Med.

[2] Helliwell T, Muller S, Hider SL, Prior JA, Richardson JC, Mallen CD (2018). Challenges of diagnosis and management of giant cell arteritis in general practice: a multimethods study. BMJ Open.

[3] Michailidou D, Zhang T, Stamatis P, Ng B (2022). Risk of venous and arterial thromboembolism in patients with giant cell arteritis and/or polymyalgia rheumatica: a veterans health administration population-based study in the United States. J Intern Med.

[4] Chan FLY, Lester S, Whittle SL, Hill CL (2019). The utility of ESR, CRP and platelets in the diagnosis of GCA. BMC Rheumatol.

[5] Simon S, Ninan J, Hissaria P (2021). Diagnosis and management of giant cell arteritis: major review. Clin Exp Ophthalmol.

[6] van Sleen Y, Graver JC, Abdulahad WH (2019). Leukocyte dynamics reveal a persistent myeloid dominance in giant cell arteritis and polymyalgia rheumatica. Front Immunol.

[7] Nadkarni S, Dalli J, Hollywood J, Mason JC, Dasgupta B, Perretti M (2014). Investigational analysis reveals a potential role for neutrophils in giant-cell arteritis disease progression. Circ Res.

[8] Oh LJ, Wong E, Gill AJ, McCluskey P, Smith JEH (2018). Value of temporal artery biopsy length in diagnosing giant cell arteritis. ANZ J Surg.

[9] Hunder GG, Bloch DA, Michel BA (1990). The American College of Rheumatology 1990 criteria for the classification of giant cell arteritis. Arthritis Rheum.

[10] Mackie SL, Dejaco C, Appenzeller S (2020). British Society for Rheumatology guideline on diagnosis and treatment of giant cell arteritis. Rheumatology.

[11] Melville AR, Donaldson K, Dale J, Ciechomska A (2022). Validation of the Southend giant cell arteritis probability score in a Scottish single-centre fast-track pathway. Rheumatol Adv Pract.

[12] Michailidou D, Duvvuri B, Kuley R (2022). Neutrophil activation in patients with anti-neutrophil cytoplasmic autoantibody-associated vasculitis and large-vessel vasculitis. Arthritis Res Ther.

[13] Oh LJ, Wong E, Andrici J, McCluskey P, Smith JEH, Gill AJ (2018). Full blood count as an ancillary test to support the diagnosis of giant cell arteritis. Intern Med J.

[14] Weiss LM, Gonzalez E, Miller SB, Agudelo CA (1995). Severe anemia as the presenting manifestation of giant cell arteritis. Arthritis Rheum.

[15] Butendieck R, Calamia K, Sandin A (2022). A study of temporal artery biopsy for the diagnosis of giant cell arteritis. Clin Rheumatol.

[16] Navahi RA, Chaibakhsh S, Alemzadeh SA, Aghdam KA (2021). The adequate number of histopathology cross-sections of temporal artery biopsy in establishing the diagnosis of giant cell arteritis. J Ophthalmic Vis Res Jan-Mar.

[17] Narváez J, Bernad B, Roig-Vilaseca D (2007). Influence of previous corticosteroid therapy on temporal artery biopsy yield in giant cell arteritis. Semin Arthritis Rheum.

